# Effectiveness of Intensive Cardiac Rehabilitation in High-Risk Patients with Cardiovascular Disease in Real-World Practice

**DOI:** 10.3390/nu13113883

**Published:** 2021-10-29

**Authors:** Iwona Świątkiewicz, Salvatore Di Somma, Ludovica De Fazio, Valerio Mazzilli, Pam R. Taub

**Affiliations:** 1 Division of Cardiovascular Medicine, University of California San Diego, La Jolla, CA 92037, USA; ptaub@health.ucsd.edu; 2 Department of Cardiology and Internal Medicine, Nicolaus Copernicus University in Toruń, Collegium Medicum in Bydgoszcz, 85-094 Bydgoszcz, Poland; 3 Department of Medical-Surgery Sciences and Translational Medicine, Sapienza University of Rome, 00189 Rome, Italy; salvatore.disomma@uniroma1.it (S.D.S.); defazio.1638451@studenti.uniroma1.it (L.D.F.); mazzilli.1626463@studenti.uniroma1.it (V.M.)

**Keywords:** cardiovascular disease, cardiovascular risk factors, cardiometabolic risks, obesity, secondary prevention, lifestyle intervention, cardiac rehabilitation, outcomes, plant-based diet, specialized diet, coronary artery disease

## Abstract

Structured lifestyle interventions through cardiac rehabilitation (CR) are critical to improving the outcome of patients with cardiovascular disease (CVD) and cardiometabolic risk factors. CR programs’ variability in real-world practice may impact CR effects. This study evaluates intensive CR (ICR) and standard CR (SCR) programs for improving cardiometabolic, psychosocial, and clinical outcomes in high-risk CVD patients undergoing guideline-based therapies. Both programs provided lifestyle counseling and the same supervised exercise component. ICR additionally included a specialized plant-based diet, stress management, and social support. Changes in body weight (BW), low-density lipoprotein cholesterol (LDL-C), and exercise capacity (EC) were primary outcomes. A total of 314 patients (101 ICR and 213 SCR, aged 66 ± 13 years, 75% overweight/obese, 90% coronary artery disease, 29% heart failure, 54% non-optimal LDL-C, 43% depressive symptoms) were included. Adherence to ICR was 96% vs. 68% for SCR. Only ICR resulted in a decrease in BW (3.4%), LDL-C (11.3%), other atherogenic lipids, glycated hemoglobin, and systolic blood pressure. Both ICR and SCR increased EC (52.2% and 48.7%, respectively) and improved adiposity indices, diastolic blood pressure, cholesterol intake, depression, and quality of life, but more for ICR. Within 12.6 ± 4.8 months post-CR, major adverse cardiac events were less likely in the ICR than SCR group (11% vs. 17%), especially heart failure hospitalizations (2% vs. 8%). A comprehensive ICR enhanced by a plant-based diet and psychosocial management is feasible and effective for improving the outcomes in high-risk CVD patients in real-world practice.

## 1. Introduction

Cardiovascular disease (CVD) is the main cause of morbidity and mortality, and the coronary artery disease (CAD) is a leading cause of death [1,2,3]. The burden of CVD continues to increase due to the high prevalence of cardiovascular (CV) risk factors such as obesity, elevated atherogenic lipids and blood pressure (BP), type 2 diabetes mellitus (T2D), an inadequate diet, low physical activity, chronic inflammation, and smoking [1,2,3,4,5]. Chronic stress, anxiety, and depression were also found as independent CV risk factors [6,7,8].

The management of high-risk CVD patients in real-world practice is challenging yet critical given increased morbidity and mortality [1,2,3]. Secondary prevention modalities including evidence-based pharmacotherapy and comprehensive risk factor management such as reducing body weight (BW) and low-density lipoprotein cholesterol level (LDL-C), controlling BP, increasing physical activity, and optimizing an unhealthy diet improve the outcomes of patients with established CVD [9,10,11,12,13,14,15]. Cardiac rehabilitation (CR) is recommended as an integral component of secondary prevention [16,17,18,19,20,21]. Exercise training remains the key element of CR; however, comprehensive structured lifestyle interventions are recommended [6,16,21,22]. Diet/nutritional and physical activity counseling, along with weight control, lipid and BP management, and psychosocial interventions are considered as the core components of modern CR programs [21].

Although beneficial effects of CR in CVD patients were demonstrated previously, mostly by small-scale clinical trials and metanalyses, there remains considerable uncertainty regarding the effectiveness of CR in real-world practice, especially in the era of modern evidence-based therapies [22,23,24,25,26]. CR programs vary considerably in intensity, duration, modalities, and delivery. It is unclear how these variations impact clinical benefits. The feasibility and effectiveness of different types of CR programs have not been well studied, especially in high-risk CVD populations [22,23,24,25,26,27]. Also, comparative analyses of exercise-based standard CR (SCR) and multi-component intensive CR (ICR) in patients with various types of CVD, which could aid in identifying a specific CR program most likely to benefit CVD patients, are lacking.

This study aims at evaluating a comprehensive multifactorial ICR program and exercise-based SCR program in real-world practice in terms of feasibility and improving cardiometabolic outcomes, depression, and health-related quality of life (QoL), as well as reducing major adverse cardiac events (MACEs) in long-term follow-up in a broad spectrum of CVD patients undergoing guideline-based therapies.

## 2. Materials and Methods

### 2.1. Study Design and Participants

We performed a retrospective longitudinal cohort study of CVD patients undergoing outpatient ICR and SCR programs at the Cardiac Rehabilitation and Wellness Center University of California San Diego (UCSD) from 1 January 2018 to 12 February 2019. Medical records were analyzed for baseline characteristics, cardiometabolic, and psychosocial outcomes, and long-term MACEs. Both programs were carried out in the same UCSD facility under the supervision of the same multidisciplinary team with experience in CR delivery, and was led by a cardiologist. The choice of CR program (ICR vs. SCR) was based on patient preference. The study was conducted in accordance with the Declaration of Helsinki. Approval from the ethics committee of UCSD Human Research Protections Program was obtained (IRB 190538/3/27/2019). Written informed consent was waived given the retrospective nature of the study.

Participants were adults aged ≥18 years who underwent CR and had medical data available to review. Patients were referred for CR by their cardiologists if they had at least one of the following: acute myocardial infarction (MI) within the last 12 months, stable angina, coronary artery bypass grafting (CABG), percutaneous coronary intervention (PCI), heart valve repair/replacement, stable chronic systolic heart failure (HF), a heart transplant, and peripheral artery disease. The diagnosis of stable angina was made if symptoms continued to occur >1 year after diagnosis or revascularization, or vasospastic/microvascular disease was diagnosed. Stable chronic systolic HF was defined as the left ventricular ejection fraction (LVEF) ≤ 35% and NYHA class II-IV symptoms despite optimal therapy for ≥6 weeks.

The ICR program (specifically, the Ornish program, Sharecare, Inc., Atlanta, GA, USA) included a structured class model twice a week (4 hours) over nine weeks (18 sessions, 72 hours in total) comprising supervised exercise training, a specialized plant-based diet, education on nutrition and a healthy lifestyle, stress management, and social support. The ICR was provided by a multidisciplinary team (cardiologist, registered nurse, exercise physiologist, registered dietitian, behavioral health specialist, and clinical psychologist). During each session, participants shared a nutritious, plant-based meal during a cooking lesson and learned about lifestyle behavior. Nutritional counselling about a specific plant-based diet along with written instructional materials were provided to patients. A whole food, low-fat, low in refined carbohydrates, nutritionally adequate, plant-based diet (consisting of fruits, vegetables, whole grains, legumes, and soy products) without caloric restriction was recommended. Specific recommendations included ≤10% of total calorie intake as fat (polyunsaturated/saturated fat ratio > 1, ~25 g of fat supplemented with 3 g/day of fish oil or plankton-based omega 3 fatty acids), 15–20% of calories as protein (average ~55 g of protein for women and ~65 g for men), 70–75% of calories as carbohydrates (predominantly complex carbohydrates, refined carbohydrates limited to ≤2 servings), cholesterol intake ≤10 mg/day, sodium intake ≤2.3 g/day (<1.5 g in hypertensive patients), no caffeine, and alcohol <2 units/day. No animal products were allowed except egg white and one cup per day of non-fat milk or yogurt. The diet was nutritionally adequate and met the recommended daily allowances for all nutrients except vitamin B12, which was supplemented. Stress management included stretching exercises, breathing techniques, meditation, progressive relaxation, and imagery. Social support was provided through regular group meetings led by a clinical psychologist.

The SCR program involved exercise and educational sessions on nutrition and healthy lifestyle (1 h) 3 days per week over 12 weeks (36 sessions, 36 h in total). Healthy food choices were recommended, such as saturated fatty acids <10% of total energy intake (through replacement by polyunsaturated fatty acids), ≥200 g of fruit/day (2–3 servings), ≥200 g of vegetables/day (2–3 servings), fish 1–2 times/week (one of which to be oily), <5 g of salt/day, 30–45 g of fiber/day (preferably from wholegrain products), limited sugar-sweetened soft drinks, and alcoholic beverages ≤2 units/day (20 g/day of alcohol) for men and 1 unit/day (10 g/day of alcohol) for women [16].

Structured and supervised exercise included regular, moderate aerobic and resistance/strength training. The exercise component was the same in both CR programs. Patients were individually prescribed exercise levels (typically walking) according to baseline exercise treadmill testing (ETT) results. The target training heart rate (HR) was 50–70% of the HR, at which symptoms and/or ECG changes occurred or 55–70% of predicted age-adjusted maximum HR based on patient conditioning level.

Adherence to the CR program was defined as a percentage of sessions that participants attended during the CR program.

### 2.2. Cardiometabolic Outcomes

Cardiometabolic outcomes were assessed at the entry and after completion of the CR program.

Metabolic outcomes included BW, body mass index (BMI), waist circumference (WC), body fat percentage (BF), visceral fat rating (VF), glycated hemoglobin (HbA1c), and lipid levels. BW and WC were measured in a fasted state using a digital scale and anthropometric tape, respectively. Body composition was analyzed using a bioelectrical impedance method (InBody 570 analyzer, Cerritos, CA, USA). Overweight, obesity, abdominal obesity, and excessive VF were defined as a BMI of ≥25 kg/m^2^, BMI ≥ 30 kg/m^2^, WC ≥ 102 cm in men and ≥88 cm in women, and VF score > 12, respectively.

Laboratory tests including total cholesterol (TC), LDL-C, non-high-density lipoprotein cholesterol (non-HDL-C), high-density lipoprotein cholesterol (HDL-C), and triglycerides (TG) were performed in serum samples in a certified analytical laboratory. Blood samples for lipids were drawn after a 12-hour fast. According to current guidelines during the study, the reduction of LDL-C < 70 mg/dL was considered as the target LDL-C post-CR [13,14]. The LDL particle number (LDL-P) was measured by LipoFit NMR (ARUP Laboratories, Salt Lake City, UT, USA).

Dietary cholesterol intake was assessed in both groups based on the analysis of a diet diary which was completed by patients at the baseline and at the completion of the CR program to assess nutrient intake and dietary adherence. In the ICR group, data on dietary fat and fiber intake at the baseline and at the completion of the ICR program were also available for analysis.

Cardiac outcomes included peak exercise capacity (EC), systolic and diastolic BP, and resting HR. The peak EC was quantified as metabolic equivalents (METS) during ETT that was performed according to standardized protocols, mainly with the Bruce protocol. Patients exercised as long as possible depending on the conditioning level to achieve at least 70–85% of the predicted age-adjusted maximum HR or until symptoms (such as dyspnea, fatigue, and chest pain), 1 mm ST depression in ECG, abnormal BP response, or ventricular ectopy occurred. BP and HR measurements were done after a 5-min rest, and resulted in an output that was an average of three readings 1–2 minutes apart. Transthoracic echocardiography was performed at the CR entry to assess LVEF.

### 2.3. Psychosocial Outcomes

According to the standard medical care at the Cardiac Rehabilitation and Wellness Center at UCSD, different questionnaires for psychosocial outcomes are used for different CR programs. For depression and QoL assessment in the ICR group, the Center for Epidemiologic Studies Depression Scale (CES-D) and 36-Item Short Form Health Survey (SF-36) questionnaires were completed at CR entry and completion. Similarly, for the SCR group, the Patient Health Questionnaire-9 (PHQ-9) and Quality of Life Scale (QOLS) were used.

The CES-D scores of 10–15 points and ≥16 points indicate mild and significant depression, respectively. The PHQ-9 scores of ≥10 and ≥5 indicate major depression disorder and at least mild depression, respectively. Higher SF-36 scores reflect better mental and physical self-rated health. A higher QOLS score indicates better self-rated QoL.

### 2.4. Major Adverse Cardiac Events

The incidence of MACEs in long-term follow-up was evaluated where MACEs included all-cause death, non-fatal MI, hospitalization for unstable angina, PCI, CABG, peripheral artery revascularization, ischemic stroke, hospitalization for HF (HFH), heart valve repair or replacement, and heart transplant or left ventricular assist device implantation. Total MACE was defined as the composite of all of the above.

Data on MACEs were acquired for the period from the completion of the CR program through 30 December 2019. MACEs were identified and adjudicated based on the medical records (including the record of hospitalizations, clinic visits, and medical notes) by two cardiologists. Non-fatal MI was defined using the fourth universal definition of MI. HFH was defined as post-CR hospital admission due to new or increasing HF symptoms and signs in combination with a change in treatment to improve HF, including the parenteral use of a diuretic.

### 2.5. Primary and Exploratory Study Outcomes

Post-CR changes in mean BW, LDL-C, and peak EC were the primary study outcomes. The incidence of total MACE in the long-term follow-up was the exploratory outcome.

### 2.6. Statistical Analysis

Continuous data were presented as means ± standard deviation. The Kolmogorov-Smirnov test was performed to evaluate the assumption of normality. A paired t-test was used for testing the equality of means of continuous parameters within the ICR and SCR groups. The paired samples *t*-tests were used for comparing baseline and post-CR values. Repeated Measure Analysis of Variance (RM-ANOVA) was used for testing the differences between the groups between baseline and post-CR values. An effect size using Cohen’s *d* was used for a quantitative measure of the magnitude of post-CR change in depression and QoL. Nonparametric Spearman’s rank correlation coefficient, *r*_s_, was calculated to describe the statistical dependence between the rankings of two variables. The correlation coefficient is reported as the absolute value |*r*_s_|. The Kaplan-Meier method was used to estimate the probability of event-free survival. A two-tailed *p* < 0.05 was considered statistically significant. All statistical analyses were performed using the SPSS system version 26 (IBM, Chicago, IL, USA).

## 3. Results

Initially, 331 patients (104 ICR and 227 SCR) were screened for this study. Based on medical records, 17 patients overall were excluded; three due to a lack of CVD, 11 due to changes in CV pharmacotherapy during the study period, and three due to hospitalizations during CR. Finally, a total of 314 patients (101 ICR and 213 SCR) were included in the study.

Prior coronary revascularization, prior MI, stable angina, chronic stable systolic HF, and heart transplant were the most common indications for CR in ICR group (64%, 13%, 12%, 6%, and 3% of patients, respectively). In the SCR group, prior coronary revascularization, prior MI, chronic stable systolic HF, heart valve replacement, stable angina, and heart transplant (33%, 18%, 16%, 16%, 9%, and 5% respectively) were the most common indications.

Adherence to the CR program was 95.8 ± 9% in the ICR group vs. 68.1 ± 36.7% in the SCR (*p* < 0.001). The long-term follow-up for MACEs was 12.6 ± 4.8 months in the entire study group.

### 3.1. Baseline Clinical Characteristics

The baseline characteristics for ICR and SCR groups are displayed in Table 1 and Table 2. No differences were observed between the ICR and SCR groups in age, gender, race/ethnicity, and most biochemical parameters, as well as incidence of T2D (24% vs. 29%) and chronic kidney disease (CKD) (26% vs. 35%), which were highly prevalent in both groups. In both the ICR and SCR groups, CAD (95% vs. 88% of patients), hypertension (HTN) (66% vs. 75%), and chronic symptomatic HF (22% vs. 32%) were the most common CVD. While CAD, prior PCI, atrial fibrillation, and diagnosis of hyperlipidemia were more prevalent in ICR patients than SCR, prior non-ST-elevation MI, ischemic stroke, and heart valve replacement were more common in the SCR group. The mean LVEF was preserved at CR entry in both groups.

No differences between the groups were observed in the baseline CV risk factors such as BW (*p* = 0.564), BMI (*p* = 0.324), BF (*p*= 0.990), VF (*p* = 0.670), WC (*p* = 0.560), systolic BP (*p* = 0.076), TC (*p* = 0.877), LDL-C (*p* = 0.715), non-HDL-C (*p* = 0.722), TG (*p* = 0.132), lipoprotein (a), fasting plasma glucose, and HbA1c (*p* = 0.188), as well as peak EC (*p* = 0.068).

In both groups, patients received guideline-based pharmacotherapy that was not changed during the study period. Statins, mostly high-dose atorvastatin and rosuvastatin (~90% of patients on statin therapy), were administered to the majority of patients in both the ICR and SCR groups (90% vs. 82%). While more SCR patients than ICR patients (39% vs. 15%) received antidepressant agents, no differences in other pharmacotherapies were observed between groups.

### 3.2. Cardiometabolic Outcomes

**Figure 1:**
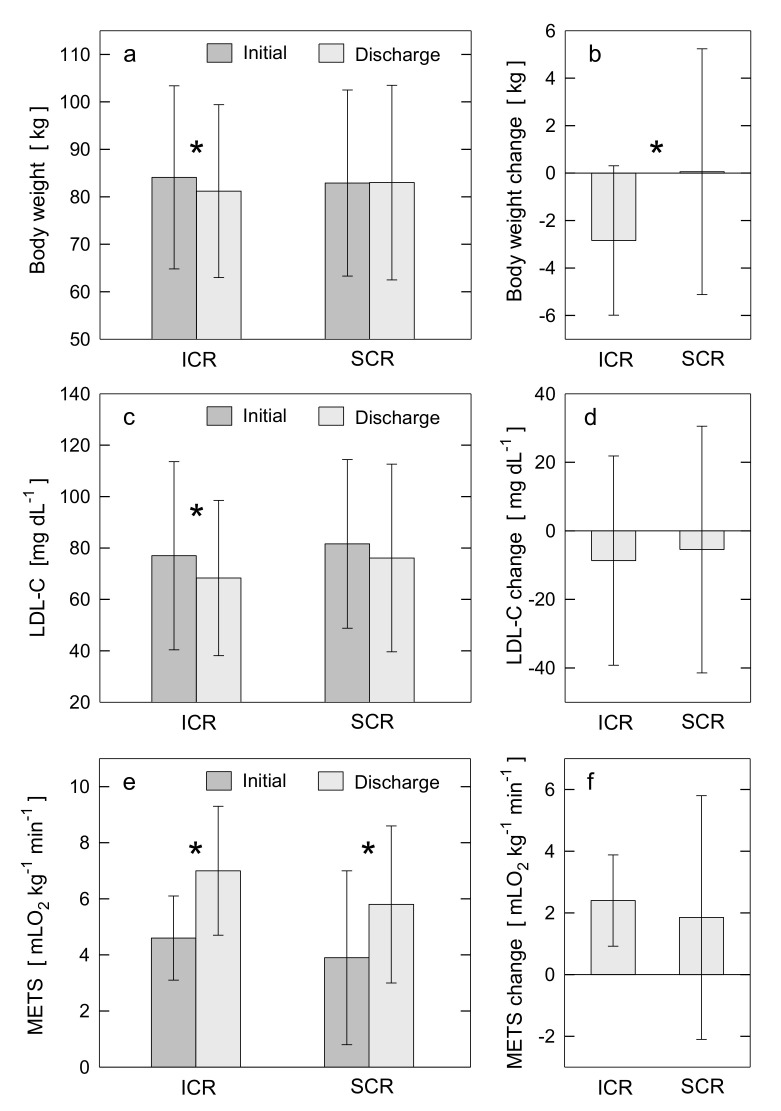
Changes in cardiometabolic outcomes between the entry (Initial) and discharge (Discharge) from the CR programs within ICR and SCR groups (**a**,**c**,**e**) and between ICR and SCR groups (**b**,**d**,**f**). (**a**,**c**,**e**) depict post-CR changes within ICR and within SCR groups in body weight, LDL-C, and peak EC quantified as METS, respectively. (**b**,**d**,**f**) depict the differences between ICR and SCR groups in post-CR changes for body weight, LDL-C, and METS, respectively. Each bar plot displays the mean value and standard deviation. The symbol * indicates the statistical significance for a given change in outcome. If symbol * is not placed, it indicates no statistical significance. For statistically significant results, the *p*-values are: panel a: *p* < 0.0001 for post-ICR change in body weight; c: *p* = 0.006 for post-ICR change in LDL-C; e: *p* < 0.0001 for post-ICR and post-SCR changes in METS; b: *p* < 0.0001 for difference in post-CR change in body weight between ICR and SCR. CR: cardiac rehabilitation; EC: exercise capacity; ICR: intensive cardiac rehabilitation; LDL-C: low-density lipoprotein cholesterol level; METS: metabolic equivalents; SCR: standard cardiac rehabilitation.

Changes in the cardiometabolic outcomes between the entry and discharge from the CR program within and between the ICR and SCR groups are shown in Table 2 and Figure 1.

At baseline, 74% of ICR patients vs. 76% of SCR (*p* = 0.615) were overweight, 25% of ICR vs. 27% of SCR (*p* = 0.643) were obese, and 54% of ICR vs. 48% of SCR (*p* = 0.528) had abdominal obesity. No differences in most cardiometabolic outcomes were observed between the groups before the CR program (see Section 3.1 and Table 1 and Table 2). Mean LDL-C was non-optimal (≥70 mg/dL) in both groups. Optimal LDL-C (<70 mg/dL) occurred in 51% of ICR patients vs. 43% of SCR (*p* = 0.225). The mean BP was in normal range for both groups; however, diastolic BP was lower in the SCR group than ICR (*p* < 0.001). The mean LDL-P (*p* = 0.049) and HR (*p* = 0.046) were higher in the SCR group than ICR. The mean cholesterol intake was higher in the ICR group than SCR (*p* < 0.001). In both groups, the cholesterol intake and fat intake (37.2 ± 8% of total calories/day for ICR vs. 31.5 ± 12% for SCR, *p* = 0.395) were higher, and fiber intake was lower than recommended [16].

The ICR program resulted in a significant decrease in BW (3.4%) (Figure 1a,b), BMI (3.5%), WC (3.3%), BF (6.0%), VF (11.6%), HbA1c (1.7%), and atherogenic lipids such as TC (6.9%), LDL-C (11.3%) (Figure 1c,d), non-HDL-C (13.4%), and LDL-P (6.5%). In addition, a decrease in systolic (4.1%) and diastolic (11.3%) BP and cholesterol intake (77.7%), and an increase in peak EC (52.2%) (Figure 1e,f) were observed. No significant changes in TG and HDL-C were found, though TG modestly decreased. As a result of ICR, dietary fat intake decreased (55.5%, *p* < 0.001) and fiber intake increased (41%, *p* < 0.001).

The greater decrease in BW post-ICR was accompanied by a greater reduction in WC (|*r*_s_| = 0.545, *p* < 0.01). A weak correlation (|*r*_s_| = 0.22–0.25, *p* < 0.05) was observed between the decrease in BW and the decrease in BF, the decrease in LDL-P, the improved level of LDL-C post-ICR, and the decrease in diastolic BP. A similar level of correlation was found between the increase in peak EC and improved level of LDL-C post-ICR, and slightly better correlation for the decrease in dietary cholesterol intake and the decrease in TC post-ICR (|*r*_s_| = 0.307, *p* < 0.01).

The SCR program resulted in a decrease in WC (1.3%), BF (3.1%), VF (7.2%), diastolic BP (3.1%), and cholesterol intake (17.6%), and an increase in peak EC (48.7%) (Figure 1e,f) and HR. No significant changes in other cardiometabolic outcomes including BW and LDL-C were observed (Figure 1a,b). One notable result from the correlation analysis is for the adherence to SCR and the increase in peak EC post-SCR (|*r*_s_| = 0.413, *p* < 0.01).

Post-CR, incidence of overweight, obesity, and abdominal obesity decreased in the ICR group. Specifically, 68% of ICR patients vs. 76% of SCR (*p* = 0.202) were overweight, 24% of ICR vs. 30% of SCR (*p* = 0.266) were obese, and 37% of ICR vs. 34% of SCR (*p* = 0.713) had abdominal obesity. Post-CR values of BW, BMI, TC, LDL-C, non-HDL-C, LDL-P, HbA1c, and systolic BP were significantly lower than baseline values only for ICR group (Figure 1a–d). In addition, compared with SCR, ICR resulted in greater improvements in WC, BF, cholesterol intake, and diastolic BP. The mean LDL-C was <70 mg/dL post-ICR only (Table 2, Figure 1c). The target LDL-C (<70 mg/dL) was achieved more frequently post-ICR than post-SCR (59% vs. 38% of patients, *p* < 0.0001). Post-CR, mean HbA1c (*p* < 0.001), cholesterol intake (*p* < 0.001) and HR (*p* = 0.001) were lower, and peak EC (*p* < 0.001) was higher in the ICR group than the SCR. Compared to the baseline, the post-ICR dietary intake of cholesterol and fat (22.4 ± 8.1% of total calories/day) was lower, while fiber intake was higher and reached the recommended target [16]. The higher adherence to ICR was accompanied by a lower level of LDL-C post-ICR (|*r*_s_| = 0.291, *p* < 0.01). Only for the ICR was a weak negative correlation found between the peak EC and both the level of LDL-C and the decrease in LDL-P post-ICR (|*r*_s_| = 0.223, *p* < 0.05).

### 3.3. Psychosocial Outcomes

Changes in the psychosocial outcomes between the entry and discharge from the CR program within and between ICR and SCR groups are shown in Table 2.

At the baseline, 40% of ICR patients vs. 45% of SCR (*p* = 0.612) and 30% of ICR vs. 14% of SCR (*p* = 0.088) reported at least mild and significant depressive symptoms, respectively.

Both CR programs resulted in a decrease in depression scores (by 48% for ICR, 34% for SCR) and increase in QoL scores (by 11–16% for ICR, 9% for SCR). The improvement in depressive symptoms was more pronounced post-ICR compared to SCR (effect size of 0.269 vs. 0.111). Also, QoL improved more post-ICR (effect size of 0.478 for physical health and 0.310 for mental health) than SCR (effect size of 0.158).

Post-CR, at least mild and significant depressive symptoms occurred in 16% of ICR patients vs. 24% of SCR (*p* = 0.691) and 5% of ICR vs. 6% of SCR (*p* = 0.687), respectively.

### 3.4. Major Adverse Cardiac Events

The incidence of MACEs in long-term follow-up for the ICR and SCR groups is summarized in Table 3. The long-term follow-up for MACEs was 13.2 ± 4.8 months in ICR group vs. 12.0 ± 4.8 in SCR (*p* = 0.038).

Over a long-term follow-up post-CR discharge, the total MACE occurred in 48 patients (15% of total). No significant difference in total MACE was observed between groups; however, total MACE was more likely in the SCR than ICR group (17% vs. 11%, *p* = 0.136).

The mean time between CR discharge and first MACE was 150.5 ± 139.5 days in ICR group vs. 153.9 ± 149.8 days in SCR (*p* = 0.948). CV cause was a predominant cause of death in both groups. HFH was the most common MACE in the SCR group (8%), while hospitalization for unstable angina in ICR (5%).

No differences in specific MACEs including atherosclerotic MACEs (such as MI, unstable angina, PCI, CABG, PAD-related intervention, or ischemic stroke) were observed between groups, except for HFH that occurred more frequently in the SCR group than ICR (8% vs. 2%, *p* = 0.049). In total, there were 27 HFH in the SCR group (7 patients had 2–4 HFH) and 3 HFH in ICR (1 patient had 2 HFH).

Post-CR, the occurrence of angina symptoms decreased significantly in both groups (5% of ICR vs. 6.1% of SCR patients had angina symptoms post-CR, *p* = 0.681).

**Figure 2:**
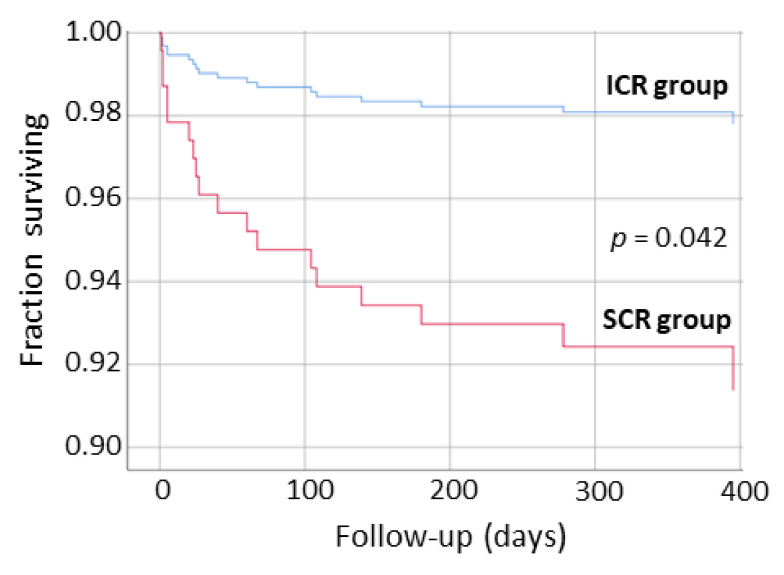
Kaplan-Meier analysis showing survival free from hospitalization for heart failure in long-term follow-up after discharge from intensive cardiac rehabilitation (ICR group) and standard cardiac rehabilitation (SCR group) program.

A Kaplan-Meier analysis showed an increased probability in long-term survival free from HFH (*p* = 0.042) for the ICR group compared to SCR (Figure 2). With regards to survival free from total MACE in a long-term follow-up, a borderline significant trend in favor of the ICR group was observed (*p* = 0.098).

### 3.5. Primary and Exploratory Study Outcomes

Regarding the primary outcomes, a significant decrease in the mean BW (2.8 kg, 3.4%, *p* < 0.0001) and mean LDL-C (8.7 mg/dL, 11.3%, *p* = 0.006), and an increase in the mean peak EC (2.4 METS, 52.2%, *p* < 0.0001) were observed post-ICR. The mean BW (increase of 0.1 kg, 0.1%, *p* = 0.878) and mean LDL-C (decrease of 5.5 mg/dL, 6.7%, *p* = 0.112) did not change significantly, while the mean peak EC significantly increased (1.9 METS, 48.7%, *p* < 0.0001) post-SCR.

No significant difference in total MACE (i.e., an exploratory outcome) was observed between the groups over the long-term follow-up post-CR; however, the total MACE was more likely in the SCR group compared to ICR (17% vs. 11% of patients, *p* = 0.136).

### 3.6. Summary of Main Results

The main findings of our study are: (1) among CVD patients undergoing CR in real-world practice, 90% had CAD with a common occurrence of other comorbidities such as HTN (66% of patients), chronic symptomatic HF (29%), T2D (27%), and CKD (32%), and CV risk factors such as increased BW (75%), non-optimal LDL-C (54%), and depressive symptoms (43%) despite guideline-based therapies; (2) adherence to ICR was high (96%), especially compared to SCR (68%); (3) ICR, but not SCR, resulted in significant improvements in most cardiometabolic outcomes such as a decrease in BW, LDL-C, other atherogenic lipids, HbA1c, and systolic BP; (4) post-ICR, target LDL-C was achieved in the majority of patients (59% vs. 38% post-SCR); (5) both ICR and SCR significantly improved peak EC; (6) both ICR and SCR decreased adiposity indices, dietary cholesterol intake, and diastolic BP, and improved depressive symptoms and QoL, but more for ICR; (7) MACEs, especially HFH, in ~1-year follow-up, were less likely post-ICR than SCR.

## 4. Discussion

Our findings support a multifactorial lifestyle intervention approach for reducing cardiometabolic risks and improving the clinical outcome of patients with CVD. A comprehensive multi-component ICR program enhanced by a plant-based diet and psychosocial management resulted in significant improvements in cardiometabolic and psychosocial outcomes, and there were trends suggesting a reduction in long-term MACEs. These improvements were greater than in the exercise-based SCR program. We demonstrate that ICR is a feasible and beneficial secondary prevention strategy in real-world clinical practice for patients who have various types of CVD, severe comorbidities, and persistent cardiometabolic risk factors despite receiving guideline-based therapies. A unique attribute of our study is the focus on evaluating the feasibility and effectiveness of standard and intensive CR programs in real-world practice. Importantly, both programs included the same exercise component and were conducted by the same multidisciplinary CR team at the same center providing high-quality CR delivery.

CR is a standard of care in CVD patients that aims to improve patient condition, affect modifiable risk factors, and prevent CVD progression or recurrence [6,16,21,22]. CR received the highest class of recommendation for various CVD therapies [16,17,18,19,20,21]. While comprehensive ICR is recommended for secondary prevention, exercise-based SCR is still the most common CR modality [6,16,21,22,23,24,26,27,28,29,30,31]. In addition, a feasibility and effectiveness of various CR programs are uncertain in real-world practice [21,22,23,24,25,26,27,28,29,30,31].

Exercise-based SCR was shown previously to reduce CV mortality and hospital admissions, and improve EC and psychosocial outcomes in patients with CAD and HF; however, effects on MI and revascularization risk and the reversal of atherosclerosis were not consistently observed [21,22,23,24,26,27,28,29,30,31]. Also, reports on the effects of SCR on modifiable risk factors in CVD patients are scarce and inconsistent, including a lack of evidence for LDL-C reduction [22,23,26,27]. In our study of high-risk patients with various types of CVD, severe comorbidities, and persistent CV risk factors, SCR did not improve most cardiometabolic markers including BW and LDL-C. Although SCR had a beneficial effect on peak EC, adiposity indices, diastolic BP, and depression and QoL, most of these improvements were smaller than for ICR. In addition, long-term MACEs, especially HFH, were more likely post-SCR than ICR.

Previous, mostly small-scale clinical trials and metanalyses demonstrated the benefits of ICR in CVD patients; however, the heterogeneity of CR programs, CR delivery, and examined populations makes it difficult to identify the optimal ICR modality [21,22,23,24,25,27,32,33,34,35,36,37]. ICR was shown to improve CV risk factors such as elevated BMI, LDL-C, and BP, as well as physical activity and dietary habits; however, significant benefits were not consistently observed [22,24,25,27,32,33,34,35,36,37,38]. Some of these improvements were maintained long term [22,24,25,27,32,33,34,35,36,37,38], especially if extended, professionally supervised, and multidisciplinary CR was implemented [35,38]. ICR programs managing more than six risk factors, and those monitoring medications to lower BP and lipids, were associated with reduction in all-cause mortality [23,27]. The multifactorial ICR was also shown to result in the regression of coronary atherosclerosis [34]. Comprehensive lifestyle intervention was demonstrated to reduce all-cause and CV mortality, MI, CV readmissions, and cerebrovascular events in patients with atherosclerotic CVD [23,27,32,35]. However, comprehensive CR (but without stress management) following MI had little or no effect on mortality, cardiac or psychological morbidity, risk factors, and QoL in a few randomized controlled trials [27,32,35,36]. Also, while comprehensive CR (including exercise training, workshops, and tailored advice) reduced the length of hospital stay and improved some CV risk factors in the heterogeneous population of patients with CVD or high CAD risk, the incidence of MACEs and QoL did not differ compared to usual care group [37].

We demonstrate that a comprehensive multifactorial ICR program resulted in significant improvements in cardiometabolic outcomes in high-risk patients with various types of CVD, including CAD and chronic HF, severe comorbidities such as T2D and CKD, and persistent CV risk factors such as elevated BW and LDL-C, despite guideline-based therapies. In addition, compared to SCR, ICR was more feasible and effective for dietary habits and psychosocial well-being, and appears to be associated with a lower incidence of long-term MACEs. We observed low rates of MACEs within a 1-year follow-up post-ICR. Importantly, these rates were lower compared to other contemporary CR studies in which CVD patients had a generally lower baseline risk of adverse CV outcome [27,32,35,36,37]. For example, in our study, total MACE was 11% vs. 16% in [35] and 31% in [37], all-cause death 1% vs. 2% [35], 4% [37] and 6% [36], non-fatal MI 1% vs. 2% [37] and 4% [36], PCI 4% vs. 9% [35] and 5% [36], CABG 0% vs. 3% [35] and 6% [36], and CV hospitalizations 10% vs. 28% [37] and 30% [36]. Our findings of post-ICR improvements in CV risk factors and a lower incidence of MACEs are consistent with previous evidence which demonstrated that even small improvements in individual risk factors lead to a significant improvement in global risk factor profiles and long-term clinical prognosis [9]. It is important to note that our study included a population of CVD patients which, in terms of an incidence of specific types of CVD and severe comorbidities, was at a higher CV risk compared to most previous ICR studies [25,33,35,36,37]. For example, the prevalence of CAD was 95% in our study vs. 58% in [37] and 49% in [25], chronic HF was 22% vs. 12% [37] and 7% [33], T2D was 24% vs. 15% [35] and 11% [36], and CKD was 26% vs. 3% [33].

The results of our study raise questions about the ICR-related mechanisms of mitigating CV risk factors, slowing or reversing CVD progression, and improving clinical outcome.

Exercise training which was provided by the ICR program in our study could benefit cardiometabolic health by improving physical fitness, glucose and lipid control, BW and composition, inflammation, and vascular and cardiac function [21,22,39,40].

The post-ICR 3% decrease in BW in our study can potentially account for beneficial effects on BP, adiposity indices such as BF and WC, and atherogenic lipids including LDL-C, which is consistent with other studies [13,14,15,41,42]. However, a decrease in BP in our study was even greater than expected from weight loss through other means [41,43]. It may indicate an importance of various mechanisms related to comprehensive lifestyle intervention rather than only BW loss for the cardiometabolic benefits of ICR.

LDL-C reduction for preventing or reversing atherosclerotic CVD is well proven and recommended in patients at-risk [9,10,11,13,14,15,16,18,19,20,21,22,44]. Any decrease in LDL-C, even moderate, contributes to reducing MACEs [10,11]. The post-ICR 11% decrease in LDL-C in our study, though moderate, allowed to achieve the target LDL-C. While a 17–22% decrease in LDL-C was reported post-ICR for more significantly elevated baseline LDL-C [25,26,33], a smaller decrease or no effect of ICR on LDL-C were also observed [27,32,35,36]. The LDL-C decrease in our study cannot be explained solely by a 3% weight loss because a 5% BW loss produces typically a 3–5% LDL-C reduction [41,43,44]. A plant-based diet, reduced cholesterol and fat intake, nutritional counselling, and exercise training could account for significant lowering LDL-C post-ICR in our study [14,15,21,22,39,40].

Post-ICR improvements in the psychosocial well-being in our study could benefit clinical outcomes. Psychosocial interventions in CAD patients were shown to improve depression, anxiety, and stress, as well as reduce cardiac mortality [6,7,8]. Compared with CR alone, comprehensive CR enhanced by stress management or mental health treatment produced a reduction in stress, which was associated with an ~50% decrease in long-term MACEs, whereas improvements in CAD biomarkers were comparable [23,33]. Comprehensive CR without stress management had no impact on risk profile and clinical outcome post-MI [27,32,36,45].

A specialized plant-based diet, high in dietary fiber, antioxidants, unsaturated fat, micronutrient content, and low in saturated fat, which was provided by ICR program in our study, was feasible and likely contributed to observed improvements in BW, atherogenic lipids such as TC, BP, and glycemic control [46,47,48]. In addition, dietary habits of CVD patients improved post-ICR. We observed a significant decrease in cholesterol intake (by 78%) and fat intake (by 56%), and an increase in fiber intake (by 41%). Previous studies demonstrated a beneficial impact of a plant-centered, high-quality diet on CVD risk factors such as atherogenic lipids, BP, and BW [46,47], as well as a risk of CVD including CAD in long-term follow-up [48,49]. Potential mechanisms of cardioprotective effects of a plant-centered diet comprising numerous beneficial compounds such as ascorbic acid, tocopherols, carotenoids, and phenolics, include antioxidant activity, inhibition of plaque formation by reducing LDL-C oxidation, platelet activation and aggregation, and anti-inflammatory effect [49]. In addition, a plant-based diet along with exercise training through various mechanisms (e.g., lowering sodium and increasing potassium intake, augmenting vasodilation and glomerular filtration rate, decreasing renin level, reducing oxidative stress, improving endothelial function, etc.) may account for a significant decrease in BP post-ICR in our study [46,47]. A reduction in systolic BP of 5 mm Hg, as observed in our study, would be expected to result in a 7%, 9%, and 14% reduction in all-cause mortality, CAD, and stroke, respectively [50].

Given that the potential difference in adherence to different CR programs is one of the real-world factors, the lower adherence to a SCR program (68%) compared to ICR (96%) could affect the results of our real-world study. Importantly, while adherence to a SCR program was relatively low, the approach implemented in the ICR program was more feasible and efficient, and resulted in a greater involvement of patients in the program. A high adherence to the ICR program along with high-quality of program delivery in our study could contribute to favorable ICR-related effects such as achieving target LDL-C, because these factors are essential to ensure expected benefits [16,21,22]. Importantly, the association between the adherence to the program and peak EC was also found for the SCR program. The effectiveness of lifestyle interventions for targeting obesity, physical inactivity, and an unhealthy diet is often limited due to a poor adherence [16,21,22,26,51,52]. ICR was shown to promote better adherence and improve the monitoring of evidence-based therapies in CVD patients, which are related to at least one-third risk reduction of all-cause mortality in CAD patients [10,21,53].

Structured lifestyle interventions in CVD patients are critical to prevent CVD progression and improve outcomes. Our findings support existing evidence that in real-world clinical practice, secondary prevention goals are not met in a substantial proportion of CVD patients. Typically, patients with CVD are a high-risk population, mostly with CAD, often with chronic HF and severe comorbidities such as T2D and CKD, and emerging CV risk factors such as obesity and elevated LDL-C, despite medical care and guideline-based therapies. Comprehensive practical solutions to urgently address cardiometabolic risks in CVD patients are desirable. The ICR program that was evaluated in this study represents the composite of feasible and efficient actions to provide tailored secondary prevention modalities in a wide spectrum of CVD patients. Our findings demonstrate that comprehensive center-based outpatient ICR is achievable, improves outcomes, and advances the management of high-risk CVD patients in real-world practice.

Despite encouraging results, various aspects associated with ICR feasibility, effectiveness, safety, and long-term sustainability of benefits in CVD patients require further evaluation and research. The referral, adherence, and standardization of CR programs need more attention [54]. Also, the mechanisms of ICR-related benefits require elucidation. Recent trials on the effectiveness of anti-inflammatory therapies for reducing the risk of CV events indicate a need for the extensive investigation of anti-inflammatory aspects of various lifestyle modifications [4,49,55,56]. Large-scale, prospective, randomized controlled trials on various ICR programs with long follow-up, also in selective populations such as women, as well as extensions of study protocols by including a comprehensive range of biomarkers (e.g., inflammatory, neuroendocrine, and oxidative stress), are needed [4,5,41,57,58,59,60]. In addition, new CR modalities such as various types of exercise training and diets, time-restricted eating, telerehabilitation, and home-based CR require further clinical research [21,22,41,61,62,63]. Future studies on lifestyle modification plans and updated guidelines and health policies are needed to alleviate long-term cardiometabolic and overall health risks in CVD patients.

This study aimed to explore real-world data on the feasibility and effectiveness of comprehensive ICR in high-risk CVD patients compared to the SCR program that is commonly applied in real-world practice. Our study included a thorough review of the medical records of high-risk patients with a broad spectrum of CVD, analysis of comprehensive set of various data, well-defined study outcomes, and long-term follow-up for MACEs. Our approach is focused on evaluating the ICR program and comparing it to the SCR program, with both programs implemented in our center. The groups of patients undergoing ICR and SCR programs were comparable in terms of baseline clinical characteristics, and both CR programs were provided in similar conditions at the same level of high-quality CR delivery. It is to be noted, however, that a further generalization of our findings on ICR effectiveness requires caution associated with sample size, 1-year period of follow-up, and lack of no-CR control group, which may be considered as a limitation. Despite some limitations, the findings of this study provide a basis for a large-scale, prospective, randomized controlled trial to determine the efficacy and sustainability of the ICR program for reducing long-term cardiometabolic risk in CVD patients.

## 5. Conclusions

The comprehensive, multifactorial lifestyle intervention approach provided by ICR, enhanced by plant-based diet and psychosocial management, had a significant impact on reducing cardiometabolic risks and improving the psychosocial well-being in high-risk patients with established CVD in real-world clinical practice. The ICR promoted weight loss and reduced adiposity, atherogenic lipids, and BP, as well as improved glycemic control, depression, QoL, and daily behaviors regarding diet and physical activity. The ICR is promising for the improvement of the long-term clinical outcome in CVD patients, especially reducing a risk for hospital readmissions. ICR appears feasible and effective as an addition to evidence-based therapies in high-risk CVD populations. ICR was also more feasible and had a greater impact on cardiometabolic risks compared to exercise-based SCR in real-world practice. Consequently, our study supports the ICR as the preferred CR modality in secondary prevention programs. Our findings also indicate a need for further clinical research to optimize ICR programs for reducing long-term cardiometabolic risks, providing tools for sustained lifestyle changes and, ultimately, improving the clinical outcome of CVD patients.

## Figures and Tables

**Table 1:** Baseline clinical characteristics of patients undergoing intensive cardiac rehabilitation (ICR group) and standard cardiac rehabilitation (SCR group).

Variable	ICR Group (*n* = 101)	SCR Group (*n* = 213)	*p*-Value between Groups
Age (years)	66.0 (±9.0)	65.0 (±15.0)	0.731
Gender (male/female) *n* (%)	69/32 (68.3/31.7)	156/57 (73.2/26.8)	0.366
Race (white/other) *n* (%)	77/24 (76.2/23.8)	154/59 (72.3/27.7)	0.460
BMI ≥25/30 kg/m^2^ *n* (%)	71 (76.3)/24 (24.7)	142 (73.6)/54 (27.3)	0.615
Coronary artery disease *n* (%)	96 (95.0)	187 (87.8)	0.044
STEMI *n* (%)	15 (14.9)	31 (14.6)	0.944
NSTEMI *n* (%)	11 (10.9)	43 (20.2)	0.041
PCI *n* (%)	66 (65.3)	107 (50.5)	0.013
CABG *n* (%)	30 (29.7)	59 (27.7)	0.713
Angina at the enrollment *n* (%)	23 (22.8)	71 (33.3)	0.056
Ischemic stroke *n* (%)	2 (2.0)	17 (8.0)	0.037
PAD *n* (%)	8 (7.9)	19 (8.9)	0.768
Hypertension *n* (%)	67 (66.3)	160 (75.1)	0.104
Heart valve replacement *n* (%)	5 (5.0)	33 (15.6)	0.007
Type 2 diabetes mellitus *n* (%)	24 (23.8)	62 (29.1)	0.321
Heart transplant *n* (%)	3 (3.0)	10 (4.7)	0.474
Chronic symptomatic heart failure *n* (%)	22 (21.8)	68 (31.9)	0.063
Chronic kidney disease *n* (%)	26 (25.7)	74 (34.7)	0.110
Atrial fibrillation *n* (%)	35 (30.4)	19 (18.8)	0.002
Hyperlipidemia *n* (%)	93 (92.1)	168 (78.9)	0.004
Smoking current/former *n* (%)	0/37 (0/36.6)	12/90 (5.6/42.3)	0.061
Family history of premature ASCVD *n* (%)	31 (30.7)	88 (41.3)	0.07
Regular aerobic exercise *n* (%)	42 (41.6)	123 (57.7)	0.024
ASA *n* (%)	94 (93.1)	183 (85.9)	0.066
Beta blocker *n* (%)	78 (77.2)	146 (68.5)	0.112
ACEI *n* (%)	28 (27.7)	65 (30.5)	0.613
ARB *n* (%)	28 (27.7)	43 (20.3)	0.142
Statin *n* (%)	91 (90.1)	175 (82.2)	0.068
Diuretic *n* (%)	28 (27.7)	82 (38.5)	0.062
Antidepressant agent *n* (%)	15 (14.9)	82 (38.7)	<0.0001
Hemoglobin (g/dL)	13.3 (±1.8)	12.5 (±2.1)	0.001
Creatinine (mg/dL)	1.15 (±1.0)	1.38 (±1.43)	0.155
Glomerular filtration rate (mL/min)	55.3 (±9.9)	52.8 (±13.4)	0.091
Leukocyte count (10^3^/µL)	7.0 (±1.9)	8.3 (±8.7)	0.164
Fasting plasma glucose (mg/dL)	105.5 (±23.2)	111.5 (±33.2)	0.065
Lipoprotein (a) (mg/dL)	47.3 (±61.5)	45.3 (±53.0)	0.833
TSH (mIU/L)	2.3 (±1.5)	2.6 (±2.1)	0.276
LVEF (%)	58.0 (±13.0)	55.0 (±16.0)	0.078

Data represent the number of patients (*n*) including the percentage of total number (%) or mean values with standard deviation (in parenthesis). Abbreviations: ACEI—angiotensin-converting enzyme inhibitor; ARB—angiotensin receptor blocker; ASA—acetylsalicylic acid; ASCVD—atherosclerotic cardiovascular disease; CABG—coronary artery bypass grafting; CVD—cardiovascular disease; HF—heart failure; ICR—intensive cardiac rehabilitation; LVEF—left ventricular ejection fraction; NSTEMI—non-ST-elevation myocardial infarction; PAD—peripheral artery disease; PCI—percutaneous coronary intervention; SCR—standard cardiac rehabilitation; STEMI—ST-elevation myocardial infarction; TSH—thyroid stimulating hormone.

**Table 2:** Changes in various parameters between the entry and discharge from cardiac rehabilitation program within and between the patient groups undergoing intensive cardiac rehabilitation (ICR group) and standard cardiac rehabilitation (SCR group).

Variable	ICR Group (*n* = 101)			SCR Group (*n* = 213)			*p*-Value between Groups
	Initial	Discharge	*p*-Value	Initial	Discharge	*p*-Value	
Body weight (kg)	84.1 (±19.3)	81.2 (±18.2)	<0.0001	82.9 (±19.6)	83.0 (±20.5)	0.878	<0.0001
BMI (kg/m^2^)	28.2 (±6.2)	27.2 (±5.8)	<0.0001	27.9 (±5.4)	27.8 (±5.3)	0.518	<0.0001
Body fat (%)	31.7 (±9.6)	29.8 (±9.8)	<0.0001	32.0 (±8.5)	31.0 (±8.7)	0.002	0.049
Visceral fat rating	12.9 (±4.9)	11.4 (±4.8)	<0.0001	12.5 (±5.1)	11.6 (±4.5)	0.001	0.163
Muscle mass (%)	67.5 (14.2)	67.5 (±13.3)	0.930	79.0 (±26.8)	72.7 (±21.1)	0.005	0.005
Waist circumference (cm)	99.6 (±15)	96.3 (±13.7)	<0.0001	100.6 (±16)	99.3 (±15.5)	0.002	0.002
Total cholesterol (mg/dL)	151.6 (±44.8)	141.2 (±36.5)	0.005	156.0 (±43.2)	149.0 (±44.5)	0.098	0.552
LDL-C (mg/dL)	77.0 (±36.6)	68.3 (±30.2)	0.006	81.6 (±32.8)	76.1 (±36.5)	0.112	0.488
LDL particle number (nmol/L)	1023.8 (±379.4)	957.3 (±362.5)	0.026	1074.3 (±561.8)	1095.3 (±277.3)	0.777	0.504
Non-HDL-C (mg/dL)	104.4 (±41.8)	90.4 (±37.9)	0.001	105.9 (±38.8)	99.5 (±41.1)	0.113	0.200
HDL-C (mg/dL)	48.5 (±14.1)	48.2 (±16.3)	0.718	49.3 (±16.5)	49.7 (±16.3)	0.637	0.558
Triglycerides (mg/dL)	131.1 (±59.5)	122.5 (±56.6)	0.089	125.3 (±66.5)	125.8 (±78.4)	0.937	0.265
Glycated hemoglobin (%)	5.8 (±0.9)	5.7 (±0.7)	0.025	6.4 (±1.3)	6.4 (±1.0)	0.930	0.357
Peak exercise capacity (METs)	4.6 (±1.5)	7.0 (±2.3)	<0.0001	3.9 (±3.1)	5.8 (±2.8)	<0.0001	0.177
Systolic BP (mmHg)	122.0 (±16.8)	117.0 (±16.9)	0.001	119.0 (±17.4)	116.0 (±16.4)	0.055	0.220
Diastolic BP (mmHg)	71.0 (±9.6)	63.0 (±9.5)	<0.0001	65.0 (±10.4)	63.0 (±10.2)	0.048	<0.0001
Heart rate (bpm)	69.0 (±11.0))	68.0 (±11.6)	0.752	72.0 (±13.6)	74.0 (±13.2)	0.005	0.043
CES-D score (-)	10.4 (±10.3)	5.4 (±5.7)	<0.0001	-	-	-	
SF-36 physical health score (-)	44.3 (±9.7)	51.2 (±7.8)	<0.0001	-	-	-	
SF-36 mental health score (-)	50.1 (±10.0)	55.6 (±5.4)	<0.0001	-	-	-	
PHQ-9 score (-)	-	-	-	5.0 (±4.5)	3.3 (±3.8)	0.001	
QOLS score (-)	-	-	-	22.6 (±5.1)	24.7 (±3.9)	<0.0001	
Dietary cholesterol intake (mg/day)	248.0 (±230.6)	55.3 (±67.1)	<0.0001	158.5 (±43.3)	130.6 (±44.8)	0.009	0.002
Dietary fat intake (g/day)	71.9 (±41.4)	32.0 (±20)	<0.0001	64.1 (±26.5)	-	-	
Dietary fiber intake (g/day)	21.7 (±10.3)	30.6 (±11.4)	<0.0001	24.1 (±1.6)	-	-	

Data represent mean values with standard deviation (in parenthesis). The *p*-values in the last column are for comparison of post-CR changes in outcomes between the ICR and SCR groups. Abbreviations: BMI—body mass index; BP—blood pressure; CES-D—Center for Epidemiologic Studies Depression Scale; HDL-C—high-density lipoprotein cholesterol; LDL-C—low-density lipoprotein cholesterol; METs—metabolic equivalents; Non-HDL-C—non-high-density lipoprotein cholesterol; PHQ-9—Patient Health Questionnaire-9; QOLS—Quality of Life Scale; SF-36—36-Item Short Form Health Survey.

**Table 3:** Incidence of major adverse cardiac events in the long-term follow-up after completion of cardiac rehabilitation in patients undergoing intensive cardiac rehabilitation (ICR group) and standard cardiac rehabilitation (SCR group).

Major Adverse Cardiac Event	ICR Group (*n* = 101)	SCR Group (*n* = 213)	*p*-Value between Groups
All-cause death *n* (%) Cardiovascular death *n* (%)	1 (1.0) 1 (1.0)	5 (2.3) 3 (1.4)	0.412 0.757
Non-fatal myocardial infarction *n* (%)	1 (1.0)	4 (1.9)	0.557
Hospitalization for unstable angina *n* (%)	5 (5.0)	10 (4.7)	0.921
PCI *n* (%)	4 (4.0)	7 (3.3)	0.762
CABG *n* (%)	0 (0.0)	1 (0.5)	0.490
Peripheral artery revascularization *n* (%)	0 (0.0)	3 (1.4)	0.231
Ischemic stroke *n* (%)	0 (0.0)	0 (0.0)	
Hospitalization for heart failure *n* (%)	2 (2.0)	16 (7.5)	0.049
Heart valve repair or replacement *n* (%)	1 (1.0)	1 (0.5)	0.588
Heart transplant or LVAD implantation *n* (%)	0 (0.0)	1 (0.5)	0.490
Total MACE *n* (%)	11 (10.9%)	37 (17.4%)	0.136

Data represent the number of patients (*n*) including the percentage of total number (%). Abbreviations: CABG—coronary artery bypass grafting; LVAD—left ventricular assist device; MACE—major adverse cardiac event; PCI—percutaneous coronary intervention.

## Data Availability

The data presented in this study are available on request from the corresponding author. The data are not publicly available due to privacy restrictions.

## References

[1] GBD 2019 Diseases and Injuries Collaborators (2020). Global burden of 369 diseases and injuries in 204 countries and territories, 1990-2019: A systematic analysis for the Global Burden of Disease Study 2019. Lancet.

[2] Virani S.S., Alonso A., Benjamin E.J., Bittencourt M.S., Callaway C.W., Carson A.P., Chamberlain A.M., Chang A.R., Cheng S., Delling F.N. (2020). On behalf of the American Heart Association Council on Epidemiology and Prevention Statistics Committee and Stroke Statistics Subcommittee. Heart Disease and Stroke Statistics—2020 Update: A Report from the American Heart Association. Circulation.

[3] Khan S.S., Ning H., Wilkins J.T., Allen N., Carnethon M., Berry J.D., Sweis R.N., Lloyd-Jones D.M. (2018). Association of Body Mass Index with Lifetime Risk of Cardiovascular Disease and Compression of Morbidity. JAMA Cardiol..

[4] Świątkiewicz I., Taub P.R. (2018). The usefulness of C-reactive protein for the prediction of post-infarct left ventricular systolic dysfunction and heart failure. Kardiol. Pol..

[5] Świątkiewicz I., Magielski P., Kubica J., Zadourian A., DeMaria A.N., Taub P.R. (2020). Enhanced inflammation is a marker for risk of post-infarct ventricular dysfunction and heart failure. Int. J. Mol. Sci..

[6] Pogosova N., Saner H., Pedersen S.S., Cupples M.E., McGee H., Höfer S., Doyle F., Schmid J.P., von Känel R. (2015). Cardiac Rehabilitation Section of the European Association of Cardiovascular Prevention and Rehabilitation of the European Society of Cardiology. Psychosocial aspects in cardiac rehabilitation: From theory to practice. A position paper from the Cardiac Rehabilitation Section of the European Association of Cardiovascular Prevention and Rehabilitation of the European Society of Cardiology. Eur. J. Prev. Cardiol..

[7] Richards S.H., Anderson L., Jenkinson C.E., Whalley B., Rees K., Davies P., Bennett P., Liu Z., West R., Thompson D.R. (2017). Psychological Interventions for Coronary Heart Disease. Cochrane Database Syst. Rev..

[8] Rao A., Zecchin R., Newton P.J., Phillips J.L., DiGiacomo M., Denniss A.R., Hickman L.D. (2020). The prevalence and impact of depression and anxiety in cardiac rehabilitation: A longitudinal cohort study. Eur. J. Prev. Cardiol..

[9] Yusuf S., Hawken S., Ounpuu S., Dans T., Avezum A., Lanas F., McQueen M., Budaj A., Pais P., Varigos J. (2004). Effect of potentially modifiable risk factors associated with myocardial infarction in 52 countries (the INTERHEART study): Case-control study. Lancet.

[10] Chow C.K., Jolly S., Rao-Melacini P., Fox K.A., Anand S.S., Yusuf S. (2010). Association of Diet, Exercise, and Smoking Modification with Risk of Early Cardiovascular Events after Acute Coronary Syndromes. Circulation.

[11] Cholesterol Treatment Trialists’ (CTT) Collaborators (2010). Efficacy and safety of more intensive lowering of LDL cholesterol: A meta-analysis of data from 170000 participants in 26 randomised trials. Lancet.

[12] Smith S.C., Benjamin E.J., Bonow R.O., Braun L.T., Creager M.A., Franklin B.A., Gibbons R.J., Grundy S.M., Hiratzka L.F., Jones D.W. (2011). AHA/ACCF Secondary Prevention and Risk Reduction Therapy for Patients with Coronary and Other Atherosclerotic Vascular Disease: 2011 Update. Circulation.

[13] Piepoli M.F., Hoes A.W., Agewall S., Albus C., Brotons C., Catapano A.L., Cooney M.T., Corrà U., Cosyns B., Deaton C. (2016). 2016 European Guidelines on cardiovascular disease prevention in clinical practice: The Sixth Joint Task Force of the European Society of Cardiology and Other Societies on Cardiovascular Disease Prevention in Clinical Practice (constituted by representatives of 10 societies and by invited experts) Developed with the special contribution of the European Association for Cardiovascular Prevention & Rehabilitation (EACPR). Eur. Heart J..

[14] Grundy S.M., Stone N.J., Bailey A.L., Beam C., Birtcher K.K., Blumenthal R.S., Braun L.T., de Ferranti S., Faiella-Tommasino J., Forman D.E. (2019). 2018 Guideline on the Management of Blood Cholesterol. A Report of the American College of Cardiology/American Heart Association Task Force on Clinical Practice Guidelines. Circulation.

[15] Mach F., Baigent C., Catapano A.L., Koskinas K.C., Casula M., Badimon L., Chapman M.J., De Backer G.G., Delgado V., Ference B.A. (2020). 2019 ESC/EAS Guidelines for the management of dyslipidaemias: Lipid modification to reduce cardiovascular risk. Eur. Heart J..

[16] Piepoli M.F., Corrà U., Benzer W., Bjarnason-Wehrens B., Dendale P., Gaita D., McGee H., Mendes M., Niebauer J., Zwisler A.D. (2010). Secondary prevention through cardiac rehabilitation: From knowledge to implementation. A position paper from the Cardiac Rehabilitation Section of the European Association of Cardiovascular Prevention and Rehabilitation. Eur. J. Cardiovasc. Prev. Rehabil..

[17] Ponikowski P., Voors A.A., Anker S.D., Bueno H., Cleland J.G.F., Coats A.J.S., Falk V., González-Juanatey J.R., Harjola V.P., Jankowska E.A. (2016). 2016 ESC Guidelines for the diagnosis and treatment of acute and chronic heart failure: The Task Force for the diagnosis and treatment of acute and chronic heart failure of the European Society of Cardiology (ESC) Developed with the special contribution of the Heart Failure Association (HFA) of the ESC. Eur. Heart J..

[18] Ibanez B., James S., Agewall S., Antunes M.J., Bucciarelli-Ducci C., Bueno H., Caforio A.L.P., Crea F., Goudevenos J.A., Halvorsen S. (2018). 2017 ESC Guidelines for the management of acute myocardial infarction in patients presenting with ST-segment elevation: The Task Force for the management of acute myocardial infarction in patients presenting with ST-segment elevation of the European Society of Cardiology (ESC). Eur. Heart J..

[19] Neumann F.J., Sousa-Uva M., Ahlsson A., Alfonso F., Banning A.P., Benedetto U., Byrne R.A., Collet J.P., Falk V., Head S.J. (2019). 2018 ESC/EACTS Guidelines on myocardial revascularization. Eur. Heart J..

[20] Knuuti J., Wijns W., Saraste A., Capodanno D., Barbato E., Funck-Brentano C., Prescott E., Storey R.F., Deaton C., Cuisset T. (2020). 2019 ESC Guidelines for the diagnosis and management of chronic coronary syndromes. Eur. Heart J..

[21] Ambrosetti M., Abreu A., Corrà U., Davos C.H., Hansen D., Frederix I., Iliou M.C., Pedretti R.F., Schmid J.P., Vigorito C. (2021). Secondary prevention through comprehensive cardiovascular rehabilitation: From knowledge to implementation. 2020 update. A position paper from the Secondary Prevention and Rehabilitation Section of the European Association of Preventive Cardiology. Eur. J. Prev. Cardiol..

[22] Lo H.C., Pazargadi A., Świątkiewicz I., Taub P., Wong N.D., Amsterdam E.A., Toth P.P. (2021). Secondary Prevention and Cardiac Rehabilitation. ASPC Manual of Preventive Cardiology.

[23] Kachur S., Chongthammakun V., Lavie C.J., De Schutter A., Arena R., Milani R.V., Franklin B.A. (2017). Impact of cardiac rehabilitation and exercise training programs in coronary heart disease. Prog. Cardiovasc. Dis..

[24] Rauch B., Davos C.H., Doherty P., Saure D., Metzendorf M.I., Salzwedel A., Völler H., Jensen K., Schmid J.P., ‘Cardiac Rehabilitation Section’, European Association of Preventive Cardiology (EAPC), in cooperation with the Institute of Medical Biometry and Informatics (IMBI), Department of Medical Biometry, University of Heidelberg, and the Cochrane Metabolic and Endocrine Disorders Group, Institute of General Practice, Heinrich-Heine University, Düsseldorf, Germany (2016). The prognostic effect of cardiac rehabilitation in the era of acute revascularisation and statin therapy: A systematic review and meta-analysis of randomized and non-randomized studies—The Cardiac Rehabilitation Outcome Study (CROS). Eur. J. Prev. Cardiol..

[25] Silberman A., Banthia R., Estay I.S., Kemp C., Studley J., Hareras D., Ornish D. (2010). The Effectiveness and Efficacy of an Intensive Cardiac Rehabilitation Program in 24 Sites. Am. J. Health Prom..

[26] Benzer W., Rauch B., Schmid J.P., Zwisler A.D., Dendale P., Davos C.H., Kouidi E., Simon A., Abreu A., Pogosova N. (2017). Exercise-based cardiac rehabilitation in twelve European countries results of the European cardiac rehabilitation registry. Int. J. Cardiol..

[27] Anderson L., Oldridge N., Thompson D.R., Zwisler A.D., Rees K., Martin N., Taylor R.S. (2016). Exercise-Based Cardiac Rehabilitation for Coronary Heart Disease: Cochrane Systematic Review and Meta-Analysis. J. Am. Coll. Cardiol..

[28] van Halewijn G., Deckers J., Tay H.Y. (2017). Lessons from Contemporary Trials of Cardiovascular Prevention and Rehabilitation: A Systematic Review and Meta-Analysis. Int. J. Cardiol..

[29] Long L., Anderson L., He J., Gandhi M., Dewhirst A., Bridges C., Taylor R. (2019). Exercise-based cardiac rehabilitation for stable angina: Systematic review and meta-analysis. Open Heart.

[30] Blumenthal J.A., Babyak M.A., O’Connor C. (2012). Effects of Exercise Training on Depressive Symptoms in Patients with Chronic Heart Failure. The HF-ACTION Randomized Trial. JAMA.

[31] Long L., Mordi I.R., Bridges C., Sagar V.A., Davies E.J., Coats A.J., Dalal H., Rees K., Singh S.J., Taylor R.S. (2019). Exercise-based cardiac rehabilitation for adults with heart failure. Cochrane Database Syst. Rev..

[32] Janssen V., De Gucht V., Dusseldorp E., Waure S.M. (2013). Lifestyle Modification Programmes for Patients with Coronary Heart Disease: A Systematic Review and Meta-Analysis of Randomized Controlled Trials. Eur. J. Prev. Cardiol..

[33] Blumenthal J.A., Sherwood A., Smith P.J. (2016). Enhancing Cardiac Rehabilitation with Stress Management Training: A Randomized Clinical Efficacy Trial. Circulation.

[34] Ornish D., Scherwitz L.W., Billings J.H., Brown S.E., Gould K.L., Merritt T.A., Sparler S., Armstrong W.T., Ports T.A., Kirkeeide R.L. (1998). Intensive lifestyle changes for reversal of coronary heart disease. JAMA.

[35] Giannuzzi P., Temporelli P.L., Marchioli R., Maggioni A.P., Balestroni G., Ceci V., Chieffo C., Gattone M., Griffo R., Schweiger C. (2008). Global secondary prevention strategies to limit event recurrence after myocardial infarction: Results of the GOSPEL study, a multicenter, randomized controlled trial from the Italian Cardiac Rehabilitation Network. Arch. Intern. Med..

[36] West R.R., Jones D.A., Henderson A.H. (2012). Rehabilitation after myocardial infarction trial (RAMIT): Multi-centre randomised controlled trial of comprehensive cardiac rehabilitation in patients following acute myocardial infarction. Heart.

[37] Zwisler A.D., Soja A.M., Rasmussen S., Frederiksen M., Abedini S., Appel J., Rasmussen H., Gluud C., Iversen L., Sigurd B. (2008). Hospital-based comprehensive cardiac rehabilitation versus usual care among patients with congestive heart failure, ischemic heart disease, or high risk of ischemic heart disease: 12-month results of a randomized clinical trial. Am. Heart J..

[38] Reich B., Benzer W., Harpf H., Hofmann P., Mayr K., Ocenasek H., Podolsky A., Pokan R., Porodko M., Puelacher C. (2020). Efficacy of extended, comprehensive outpatient cardiac rehabilitation on cardiovascular risk factors: A nationwide registry. Eur. J. Prev. Cardiol..

[39] Kränkel N., Bahls M., Van Craenenbroeck E.M., Adams V., Serratosa L., Solberg E.E., Hansen D., Dörr M., Kemps H. (2019). Exercise training to reduce cardiovascular risk in patients with metabolic syndrome and type 2 diabetes mellitus: How does it work?. Eur. J. Prev. Cardiol..

[40] Kemps H., Kränkel N., Dörr M., Moholdt T., Wilhelm M., Paneni F., Serratosa L., Solberg E.E., Hansen D., Halle M. (2019). Exercise training for patients with type 2 diabetes and cardiovascular disease: What to pursue and how to do it. A Position Paper of the European Association of Preventive Cardiology (EAPC). Eur. J. Prev. Cardiol..

[41] Świątkiewicz I., Woźniak A., Taub P.R. (2021). Time-Restricted Eating and Metabolic Syndrome: Current Status and Future Perspectives. Nutrients.

[42] Ackermann R.T., Liss D.T., Finch E.A., Schmidt K.K., Hays L.M., Marrero D.G., Saha C.A. (2015). Randomized Comparative Effectiveness Trial for Preventing Type 2 Diabetes. Am. J. Public Health.

[43] Jacobson T.A., Maki K.C., Orringer C.E., Jones P.H., Kris-Etherton P., Sikand G., La Forge R., Daniels S.R., Wilson D.P., Morris P.B. (2015). NLA Expert Panel. National Lipid association recommendations for patient-centered management of dyslipidemia: Part 2. J. Clin. Lipidol..

[44] Zomer E., Gurusamy K., Leach R., Trimmer C., Lobstein T., Morris S., James W.P., Finer N. (2016). Interventions that cause weight loss and the impact on cardiovascular risk factors: A systematic review and meta-analysis. Obes. Rev..

[45] Rutledge T., Redwine L.S., Linke S.E., Mills P.J. (2013). A Meta-Analysis of Mental Health Treatments and Cardiac Rehabilitation for Improving Clinical Outcomes and Depression among Patients with Coronary Heart Disease. Psychosom. Med..

[46] Shridhar K., Dhillon P.K., Bowen L., Kinra S., Bharathi A.V., Prabhakaran D., Reddy K.S., Ebrahim S. (2014). Indian Migration Study Group. The association between a vegetarian diet and cardiovascular disease (CVD) risk factors in India: The Indian Migration Study. PLoS ONE.

[47] Yokoyama Y., Nishimura K., Barnard N.D., Misa T., Makoto W., Akira S., Tomonori O., Yoshihiro M. (2014). Vegetarian Diets and Blood Pressure: A Meta-analysis. JAMA Intern. Med..

[48] Satija A., Bhupathiraju S.N., Spiegelman D., Chiuve S.E., Manson J.E., Willett W., Rexrode K.M., Rimm E.B., Hu F.B. (2017). Healthful and Unhealthful Plant-Based Diets and the Risk of Coronary Heart Disease in U.S. Adults. J. Am. Coll. Cardiol..

[49] Choi Y., Larson N., Steffen L.M., Schreiner P.J., Gallaher D.D., Duprez D.A., Shikany J.M., Rana J.S., Jacobs D.R. (2021). Plant-Centered Diet and Risk of Incident Cardiovascular Disease during Young to Middle Adulthood. J. Am. Heart Assoc..

[50] Whelton P.K., He J., Appel L.J., Cutler J.A., Havas S., Kotchen T.A., Roccella E.J., Stout R., Vallbona C., Winston M.C. (2002). Primary prevention of hypertension: Clinical and public health advisory from The National High Blood Pressure Education Program. JAMA.

[51] Pérez-Martínez P., Mikhailidis D.P., Athyros V.G., Bullo M., Couture P., Covas M.I., de Koning L., Delgado-Lista J., Díaz-López A., Drevon C.A. (2017). Lifestyle recommendations for the prevention and management of metabolic syndrome: An international panel recommendation. Nutr. Rev..

[52] Heymsfield S.B., Harp J.B., Reitman M.L., Beetsch J.W., Schoeller D.A., Erondu N., Pietrobelli A. (2007). Why do obese patients not lose more weight when treated with low-calorie diets? A mechanistic perspective. Am. J. Clin. Nutr..

[53] Du L., Cheng Z., Zhang Y., Li Y., Mei D. (2017). The impact of medication adherence on clinical outcomes of coronary artery disease: A meta-analysis. Eur. J. Prev. Cardiol..

[54] Freeman A.M., Taub P.R., Lo H.C., Ornish D. (2019). Intensive Cardiac Rehabilitation: An Underutilized Resource. Curr. Cardiol. Rep..

[55] Ridker P.M., Everett B.M., Thuren T., MacFadyen J.G., Chang W.H., Ballantyne C., Fonseca F., Nicolau J., Koenig W., Anker S.D. (2017). Antiinflammatory therapy with canakinumab for atherosclerotic disease. N. Engl. J. Med..

[56] Everett B.M., Cornel J.H., Lainscak M., Anker S.D., Abbate A., Thuren T., Libby P., Glynn R.J., Ridker P.M. (2019). Anti-Inflammatory Therapy With Canakinumab for the Prevention of Hospitalization for Heart Failure. Circulation.

[57] Świątkiewicz I., Magielski P., Kubica J. (2021). C-Reactive Protein as a Risk Marker for Post-Infarct Heart Failure over a Multi-Year Period. Int. J. Mol. Sci..

[58] Szewczyk-Golec K., Woźniak A., Reiter R.A. (2015). Inter-relationship of the chronobiotic, melatonin, with leptin and adiponectin: Implications for obesity. J. Pineal Res..

[59] Szewczyk-Golec K., Rajewski P., Gackowski M., Mila-Kierzenkowska C., Wesołowski R., Sutkowy P., Pawłowska M., Wozniak A. (2017). Melatonin supplementation lowers oxidative stress and regulates adipokines in obese patients on a calorie-restricted diet. Oxid. Med. Cell Longev..

[60] Kupczyk D., Bilski R., Sokołowski K., Pawłowska M., Woźniak A., Szewczyk-Golec K. (2019). Paraoxonase 1: The Lectin-Like Oxidized LDL Receptor Type I and Oxidative Stress in the Blood of Men with Type II Obesity. Dis. Markers.

[61] Świątkiewicz I., Mila-Kierzenkowska C., Woźniak A., Szewczyk-Golec K., Nuszkiewicz J., Wróblewska J., Rajewski P., Eussen S.J.P.M., Færch K., Manoogian E.N.C. (2021). Pilot Clinical Trial of Time-Restricted Eating in Patients with Metabolic Syndrome. Nutrients.

[62] Piotrowicz E., Pencina M.J., Opolski G., Zareba W., Banach M., Kowalik I., Orzechowski P., Szalewska D., Pluta S., Glówczynska R. (2020). Effects of a 9-Week Hybrid Comprehensive Telerehabilitation Program on Long-term Outcomes in Patients with Heart Failure: The Telerehabilitation in Heart Failure Patients (TELEREH-HF) Randomized Clinical Trial. JAMA Cardiol..

[63] Thomas R.J., Beatty A.L., Beckie T.M., Brewer L.C., Brown T.M., Forman D.E., Franklin B.A., Keteyian S.J., Kitzman D.W., Regensteiner J.G. (2019). Home-Based Cardiac Rehabilitation: A Scientific Statement from the American Association of Cardiovascular and Pulmonary Rehabilitation, the American Heart Association, and the American College of Cardiology. J. Am. Coll. Cardiol..

